# Remote Patient Monitoring and Telemedicine in Neonatal and Pediatric Settings: Scoping Literature Review

**DOI:** 10.2196/jmir.9403

**Published:** 2018-12-20

**Authors:** Farzan Sasangohar, Elise Davis, Bita A Kash, Sohail R Shah

**Affiliations:** 1 Industrial and Systems Engineering Department of Environmental and Occupational Health Texas A&M University College Station, TX United States; 2 National Science Foundation Center for Health Organization Transformation Department of Health Policy and Management Texas A&M University College Station, TX United States; 3 Houston Methodist Hospital Center for Outcomes Research Houston, TX United States; 4 Division of Pediatric Surgery Texas Children's Hospital Houston, TX United States; 5 Department of Surgery Baylor College of Medicine Houston, TX United States

**Keywords:** neonatal, pediatric, remote patient monitoring, telehealth, telemedicine

## Abstract

**Background:**

Telemedicine and telehealth solutions are emerging rapidly in health care and have the potential to decrease costs for insurers, providers, and patients in various settings. Pediatric populations that require specialty care are disadvantaged socially or economically or have chronic health conditions that will greatly benefit from results of studies utilizing telemedicine technologies. This paper examines the emerging trends in pediatric populations as part of a systematic literature review and provides a scoping review of the type, extent, and quantity of research available.

**Objective:**

This paper aims to examine the role of remote patient monitoring (RPM) and telemedicine in neonatal and pediatric settings. Findings can be used to identify strengths, weaknesses, and gaps in the field. The identification of gaps will allow for interventions or research to improve health care quality and costs.

**Methods:**

A systematic literature review is being conducted to gather an adequate amount of relevant research for telehealth in pediatric populations. The fields of RPM and telemedicine are not yet very well established by the health care services sector, and definitions vary across health care systems; thus, the terms are not always defined similarly throughout the literature. Three databases were scoped for information for this specific review, and 56 papers were included for review.

**Results:**

Three major telemedicine trends emerged from the review of 45 relevant papers—RPM, teleconsultation, and monitoring patients within the hospital, but without contact—thus, decreasing the likelihood of infection or other adverse health effects.

**Conclusions:**

While the current telemedicine approaches show promise, limited studied conditions and small sample sizes affect generalizability, therefore, warranting further research. The information presented can inform health care providers of the most widely implemented, studied, and effective forms of telemedicine for patients and their families and the telemedicine initiatives that are most cost efficient for health systems. While the focus of this review is to summarize some telehealth applications in pediatrics, we have also presented research studies that can inform providers about the importance of data sharing of remote monitoring data between hospitals. Further reports will be developed to inform health systems as the systematic literature review continues.

## Introduction

The United States Department of Health and Human Services defines *telehealth* as the “use of electronic information and telecommunication technologies to support and promote long-distance clinical health care, patient and professional health-related education, public health and health administration” [1]. The Agency for Healthcare Research and Quality classifies telehealth into 3 distinct categories: (1) real-time video telehealth between the patient and a health care professional; (2) store and forward telehealth, such as the sharing of medical images or data between providers; and (3) home monitoring telehealth, involving the use of telehealth to remotely monitor patients and their health status, also known as remote patient monitoring (RPM) [2]. While telehealth is used for both clinical and nonclinical applications, the term *telemedicine* is used more exclusively for clinical applications or to diagnose and treat patients [3]. Various telemedicine technologies are emerging in health care very rapidly, and some of them can potentially be cost and time saving for patients and providers as well as offer improved quality of care. Historically, telemedicine techniques and technologies have been utilized by health systems within acute care settings and patient homes most commonly to improve access to care and monitor those with the greatest need. Technologies vary in terms of cost, patient adherence and utility, effectiveness, implementation success, desired health outcomes, and impact on capacity. Pediatric patients who often lack access to specialty pediatric care are socioeconomically disadvantaged or have chronic medical needs that may especially benefit from telemedicine. There is a need to identify and describe those telemedicine devices and techniques aimed at pediatric populations that are most promising in lowering costs of care, improving patient and family experience, decreasing time spent traveling, and increasing care capacity in hospitals and clinics. In this research, we aimed to shed some light on some noteworthy telemedicine technologies successfully used for pediatric patient segments.

A systematic literature review is being conducted to examine the technologies that are currently used in health systems to effectively provide telemedicine coverage for pediatric patients from remote locations. In this paper, we present the results of the scoping review that provides our preliminary findings on the type, extent, and quantity of research available in the literature. While the overall study takes a comprehensive approach in terms of pediatric patient populations studied by disease category, complexity, and patient segment, this paper aims to highlight some emerging RPM and telemedicine trends in the neonatal and pediatric literature. Results from this research can provide an overview of available evidence to inform practitioners, including hospitals and clinics, as well as health technology developers and care providers about the current state of and opportunities in RPM and telemedicine.

We first discuss the steps taken and update on the progress of the comprehensive systematic review. Additionally, some key findings of innovations and emerging technologies in RPM and telemedicine capabilities for pediatric patients are presented. Incremental updates of this review are intended to reduce unintended consequences and costs that come with failing to utilize telemedicine capabilities within and between health systems in various settings.

## Methods

A systematic literature review is being conducted to gather an adequate amount of relevant research for telehealth in pediatric populations. The fields of RPM and telemedicine are not yet very well established by the health care services sector, and definitions vary across health care systems; thus, the terms are not always defined similarly throughout the literature. A preliminary search helped us to identify which terms provided the most literature on RPM and telemedicine and also helped us identify which databases to use.

A combination of search terms allowed us to obtain 4664 papers, which are relevant to pediatric RPM and telemedicine. All searches included either “child” or “pediatric” and at least one word comparable to “tele-monitoring,” “telehealth,” “telemedicine,” or “remote monitoring.” Some other important search terms were “population health” and “population management.”

**Figure 1:**
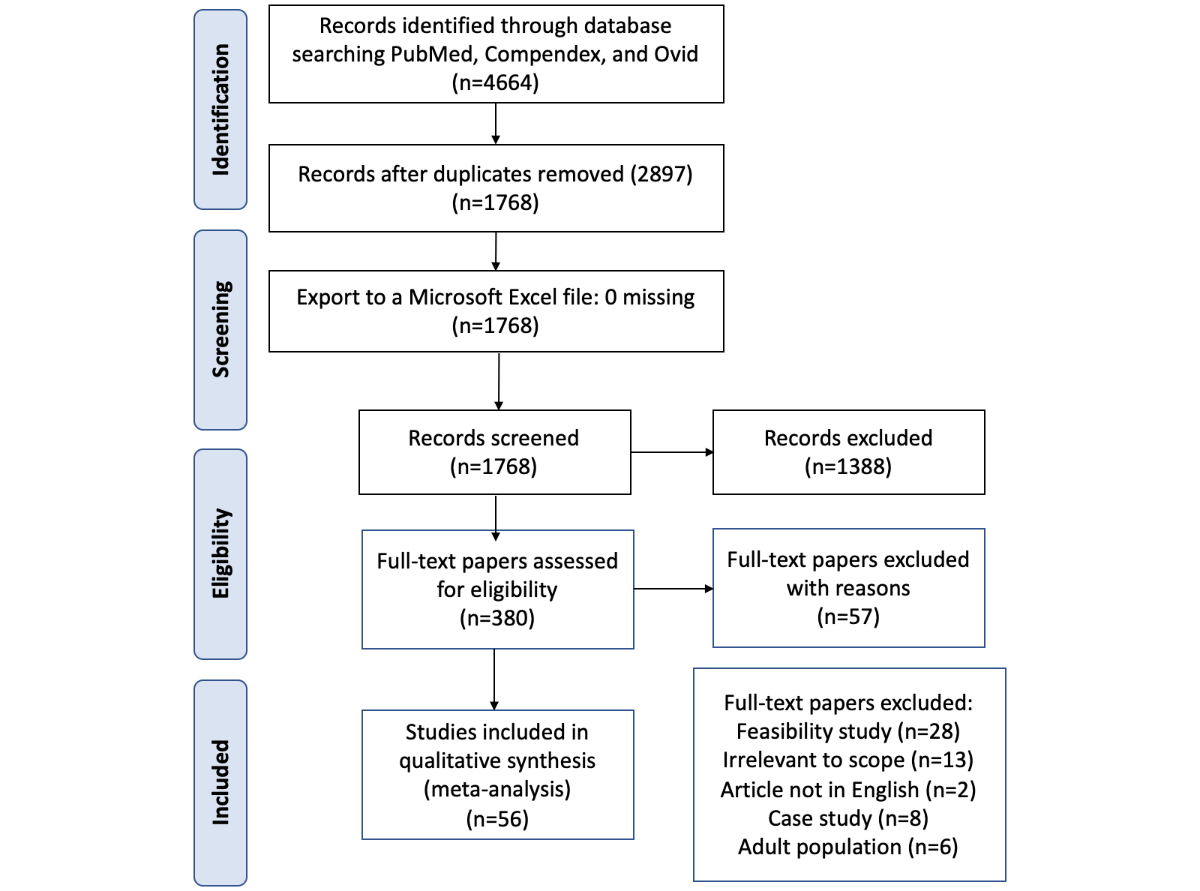
PRISMA (Preferred Reporting Items for Systematic Reviews and Meta-Analyses) diagram.

We began this search with a scope of the literature relevant to RPM and telemedicine in pediatric populations in PubMed, Compendex, and Ovid. Our search was restricted to peer-reviewed original studies published after January 1, 2008, and papers were collected between July 24 and September 2, 2016. After deleting duplicates, 1768 papers were included for an abstract review and screening. After applying the exclusion criteria, 380 papers were included for full-text review, of which 56 were selected to be included in this review. This review was conducted according to the guidelines of the Preferred Reporting Items for Systematic Review and Meta-Analyses, which visualizes the process of inclusion and exclusion of papers (Figure 1) [4]. Textbox 1 shows a brief explanation of our inclusion and exclusion criteria, respectively. A thematic analysis was then used to identify common patterns across the studies. One coder reviewed the papers and coded the RPM and telemedicine technologies used or evaluated. This paper summarizes our thematic synthesis.

**Inclusion and exclusion criteria.**

**Inclusion criteria:**

- Biometric monitoring (n=59)
- Economic benefit (n=13)
- Patient or provider satisfaction (n=55)
- Teleconsultation (n=36)
- Telediagnosis (n=120)
- Telemanagement (n=43)
- Telepresence (n=14)
- Telesupport (n=40)

**Exclusion criteria:**

- Adult population (n=224)
- Case study (n=32)
- Duplicate (n=64)
- Irrelevant (n=328)
- No specific findings (n=28)
- No original research (n=13)
- Provider-initiated contact (n=46)
- Tele-education (n=86)
- Telementoring (n=13)
- Report (n=185)
- Subjective (n=9)
- Telephone-based intervention (n=55)
- Review (n=286)

## Results

### Summary

RPM for pediatric patients can be utilized effectively in many different settings for a variety of diseases and with a variety of emerging technologies. In some cases, pediatric patients are monitored in the hospital by a physician who is in a remote location. In other cases, hospitals are using technologies to monitor patients in the hospital, but without contact, thus, decreasing the likelihood of infection or other adverse health effects. Another exciting aspect of RPM is that of monitoring patients at their home via continuous monitors or via self-uploading of patient data from a monitoring device at the home. The majority of studies we have reviewed demonstrate significant positive results, such as improved health outcomes and cost savings to patients and providers, regarding patients who are vulnerable in terms of cardiac health or diabetes. The following sections summarize the emerging themes identified in our scoping review, which examine the role of RPM technologies and provide support for their efficacy.

### Use of Continuous Glucose Monitoring

The management of type 1 diabetes in children can be challenging. Several research teams have examined the role of RPM in the management of type 1 diabetes for children, which especially helps to alert families and health professionals of hyper- and hypoglycemic critical concerns [5-10]. A key concern for these research teams was nocturnal hyper- and hypoglycemia, so glycemic levels were closely monitored throughout the day as well as during the nighttime hours.

Pena et al [9] used a glucose monitor that required patients and patient families to send glycemic information (mean blood glucose, glycated hemoglobin, and indexes of glucose volume) via email at five specific times suggested by providers throughout the day for monitoring. If any critical concerns arose, the families were contacted by the Diabetes Unit of the treating hospital via short message service text message or email. This form of RPM, which requires patients and their families to transfer data via email 5 times per day, led to adherence issues, yet it resulted in a significant decrease in glycated hemoglobin levels and overall improved metabolic control [9]. Additionally, Pena et al’s system was well accepted by parents. Unfortunately, this system was not sustainable as metabolic control returned to baseline after the study discontinued. This calls for a model for glucose monitoring that is easier for patients and families to adhere to and that emphasizes the importance of the patient-integrated care model.

The remainder of studies highlighted real-time RPM utilizing a continuous glucose monitoring (CGM) system, which simply required patients to wear a monitoring device. Three systems used CGM in association with an insulin pump so that alarms were triggered when glycemic values were critical, but insulin pumps were used to treat the critical values while the alarm was working to alert both parents and remote clinician teams of the concern [6-8]. One system did not use the insulin pump with CGM but did use alarms to alert caregivers and clinician teams in a remote setting of any critical values, thus, allowing children to be treated with appropriate levels of insulin as needed by parents or caregivers [5]. In all cases, if parents or caregivers did not respond, the remote clinician teams were available 24/7 to attempt further contact to alert caregivers of the critical values in children with type 1 diabetes.

The real-time CGM systems were able to shorten the length of hypoglycemic events in children, thus, preventing any adverse health outcomes associated with hypoglycemic events [5]. Patients and family members felt comfortable using these systems, felt that they were easy to use and understand, expressed that they would recommend the system to other families, and felt a sense of comfort knowing there was a clinician team available for backup throughout the day and at night [8]. Overall, CGM systems improved diabetes management success, and there were no safety issues identified throughout any of the studies mentioned [5-10].

### Home Monitoring of Cardiovascular Implantable Devices

Cardiovascular implantable devices are increasingly being used in the pediatric population as a method of long-term RPM [11]. A variety of research studies have examined the role of RPM with implantable devices in decreasing the incidence of adverse cardiac events [11-14]. In these studies, patients with newly implanted cardiac devices were followed either prospectively or retrospectively via RPM and compared with patients with the same devices who were monitored traditionally. All 4 studies highlighted here used automated data sent from patients to a cardiac or pacemaker care center. At the cardiac care center, data were analyzed by a cardiac physician or care nurse and contact to patients and families was initiated via the internet, telephone, or short message service text messaging depending on the results, typically in the form of an electrocardiograph (ECG). In two cases, patients were also able to report symptoms and record specific suspected cardiac events to be sent to the cardiac care team [13,14].

Researchers found several benefits from remotely monitoring pediatric patients with implantable cardiac devices. Leoni et al [14] prevented 72 clinic visits, or an average of 2 hours and 35 minutes of transportation time, for patients by monitoring symptoms remotely and communicating effectively with patients and families. In addition, 87% of patients and families rated the remote monitoring to be “very easy to perform” in the study. Leshem-Rubinow et al [13] achieved a median time between data transmission and viewing ECG data of 7 minutes; interpretation of the ECG was accomplished by trained cardiac staff within 5 hours, and the diagnosis of cardiac events averaged at 16 hours after the data transmission. Malloy et al [11] found that RPM decreased the average number of days that patients went without physician contact, potentially decreasing adverse events. For patients on a 6-month follow-up regimen, there was a temporal gain of 134 days of physician contact, and for patients on a 3-month follow-up regimen, there was a gain of 44 days. Patients in the study by Zartner et al [12] experienced 33 pacemaker shocks that successfully terminated ventricular tachycardia, improving the overall safety and well-being of patients outside a clinical setting. All researchers found that their systems were acceptable and easy to use and had a low number of false alarms from their devices. False alarms can easily be improved with continued use of devices, and they do no harm to patients or their families [11,12].

### Mobile Robotic Telemedicine in the Neonatal Intensive Care Unit

One of the benefits of telemedicine is that it allows access to specialists and subspecialists in settings where it may not be feasible or possible. Robotic telepresence (RTP) is a form of telemedicine that allows face-to-face contact between a specialist and a patient in a hospital [15,16]. An increase in preterm deliveries and survival rates with advances in neonatal medicine have resulted in a need for neonatal intensive care units (NICUs) to staff more neonatal specialists during more hours of the day [17]. A solution to these increased pressures on NICUs is the model of RTP to monitor patients in the NICU from remote locations. RTP machines are linked to the NICU and the remote location via the internet and have synchronous bidirectional audio and visual communication capabilities with zoom and a digital camera for image capture. In addition, the video screen is able to move as per the requirement of the physician or neonatal care specialist while caring for patients. A digital stethoscope, otoscope, and pulse oximeter allow the physician to check vital signs, listen to heart and bowel sounds, and better evaluate the patient while in a remote location [15,16]. By working together with onsite nurses, offsite neonatal providers can maneuver the RTP machine on their own from a distant location, and motion sensors keep the machine from bumping into any incubators or medical equipment. Visual and audio capabilities allow remote physicians to communicate with NICU nurses and families of patients.

Garingo et al [15] studied the ability of onsite and offsite neonatologists to physically examine patients in the NICU and found that local and remote physicians had good or excellent agreement for most assessments of patients. Rincon et al [16] showed that NICU nurses felt that physicians were easily accessible via RTP and that they were adequately involved and supportive of both nurses and NICU patients and their families. In addition, nurses felt they had sufficient time to ask questions and had the resources to care for patients with the simple use of RTP. A novel benefit to RTP is that neonatologists are able to monitor NICU patients during the nighttime hours, when fewer nurses are available. Overall, RTP enhanced communication and improved access for NICU patients; furthermore, cost savings are implied with remote physician capabilities.

### Telehealth Capabilities for Remote Consultation and Diagnosis

In addition to the capabilities summarized previously, telemedicine can be used for consultations and diagnosis of health concerns from remote settings. Patients and providers can save on travel time and costs, and patients who are unable to travel will benefit from specialty physician consultation via videoconferencing. In emergent cases, physicians are able to provide timely feedback to families and patients who would otherwise have to incur a great deal of costs on ground or air ambulance [18]. When using telehealth capabilities instead of telephone or email for a consult, physicians are also able to provide more accurate diagnoses and, thus, more appropriate treatment for patients [19]. In addition, physicians are able to consult with pediatric patients via a Web camera and a high-quality television screen. This allows open communication between patients, physicians, and patient families and caregivers. The same quality of care is capable of being provided in these video consults according to previous research [18,19]. Rowell et al [18] found that 40% of pediatric patients receiving orthopedic consultations via videoconferencing were discharged after one telehealth consult and 58% of patients did not require a further in-person appointment.

Dharmar et al [19] studied the effectiveness of physicians in prescribing appropriate medications and doses to pediatric patients in critical care via telemedicine. Physicians made significantly fewer medication errors in patients who received a consult via videoconferencing compared with those who received a telephone consult or did not receive a consult at all. This was an important finding as physicians were dealing with critically ill and seriously injured pediatric patients in the emergency department.

Another study examined the role of mobile telemedicine units in low-income, inner-city neighborhoods of Rochester, New York [20]. McIntosh et al [20] used health workers with minimal training to visit acute care patients along with videoconferencing capabilities to a primary care facility. By visiting patients in their homes, health workers with video access to primary care facilities saved 30% of families a trip to the emergency department and 17% of families a trip to the urgent care clinic. Close to 90% of caregivers were highly satisfied with the service and found it to be very convenient. Furthermore, McIntosh et al [20] suggested that the creation of a sustainable plan for this service with payment models included would be highly beneficial to low-income areas in the United States.

### Telemedicine Technologies Without the Use of Remote Patient Monitoring

Some technologies discovered from the literature review are relevant to telemedicine, yet they fail to utilize the aspects of RPM. Below we discuss two such systems: closed-loop systems and noncontact heart rate monitoring.

#### Closed-Loop Systems

Both Ly et al [7] and Tauschmann et al [10] used a closed-loop monitoring system, which does not require remote monitoring or supervision by clinicians. Closed-loop insulin delivery systems use a CGM device along with an automated insulin delivery device. Patients have to calibrate their devices approximately 4 times per day with a finger prick. Overall, these closed-loop systems lower mean glucose levels and reduce the amount of time spent above target glucose levels without altering daily insulin amount. In both studies, patients and families had access to clinicians or nurses 24/7 in the case of emergencies or difficulties with the system [7,10]. In this case, clinicians are left out of the loop, yet data can easily be shared remotely and monitored in case of any emergencies.

#### Noncontact Heart Rate Monitoring of Infants in the Neonatal Intensive Care Unit

Similar to closed-loop systems, several health systems are using telemedicine in hospitals, which do not require continuous physician monitoring. This creates an opportunity for sharing data with remote locations.

Several previous researchers have documented the development of robust methods for automated computation of heart rate of infants in the NICU [21,22]. Heart rate is a critical vital sign to continually assess for infants in the NICU, but current techniques involve wearing adhesive gel patches or chest straps, which can easily cause skin irritation. For NICU patients who are especially susceptible to infection, a noncontact heart rate monitor would improve overall health and decrease stress among patients and their parents [21,22]. Aarts et al and Bal used photoplethysmography (PPG), which is inexpensive and simple to use, but typically is used as a contact device using adhesive sensors [21,22]. A recent advancement is the use of camera-based PPG, a noncontact method of remotely recording PPG signals from patients using a camera and ambient light [21,22].

Aarts et al studied patients in the NICU in California and the Netherlands through noncontact PPG with an objective of exploring potential challenges of the noncontact PPG technique [21]. A total of 19 infants were examined using noncontact PPG, which provided a good measure of heart rate for >90% of the time. The study team was able to monitor heart rate by setting up a camera approximately 1 m away from infants; the camera monitored infants either through plexiglass or with open incubators. Researchers ensured that the light within the NICU was appropriate for monitoring with the camera and there was never a need for infants to be touched, removed from incubators, or repositioned throughout the study. Recordings were taken from an undressed portion of the skin (head, arm, or thorax). The recordings from the camera were saved and transferred to a computer, where the heart rate was then obtained using pulse oximetry sensors or ECG sensors [21].

There are two major limitations to noncontact PPG as identified by Aarts et al [21]. First, to be feasible, noncontact PPG must record at a random anatomical location on the skin, and noncardiovascular events may negatively affect how PPG signals are recorded. Thus, repositioning of a limb or redistribution of venous blood could affect how heart rate is identified. Additionally, the study team was unable to obtain an appropriate signal for heart rate monitoring if the infant was squirming. To ensure infant stability, the team monitored PPG signals during kangaroo mother care, and despite the slight rocking of the infant, accurate PPG signals were recorded. It is important to remember, though, that not all infants are able to engage in kangaroo mother care, and the squirming of infants remains a limitation to the noncontact PPG technique.

Bal conducted a similar study of webcam-based PPG for heart rate and oxygen saturation of healthy infants and NICU patients in Turkey [22]. Bal avoided the issue of nonstationary infants using wavelet transform, a technique that has the ability to detect rapid changes in frequency. Instead of strictly using fluorescent lighting, Bal also used sunlight for the proper detection of PPG signals and placed subjects just 50 cm from the camera. Again, recordings were sent to a computer for further analysis using ECG. Overall, Bal was able to conclude that PPG signals were accurate in both sunlight and fluorescent light and that this method monitors heart rate and oxygen saturation accurately and safely without patient contact [22].

Contactless heart rate monitoring is important in the NICU because it can help avoid infection, thus, decreasing health care cost and stress on families. Additionally, this technique is simple, inexpensive, and effective with the appropriate parameters in place such as light and distance to the camera. Neither research mentioned was disruptive of hospital or clinician flow. The avoidance of touching and repositioning infants allows patients proper rest and development within the incubator.

## Discussion

### Principal Findings

Our scoping review showed that research on telemedicine applications for pediatric populations is limited, and of the existing research, many studies are severely limited by small sample sizes and convenience samples of participants. In addition, much of the research on telemedicine technologies for pediatrics relies on the satisfaction of parents and caregivers of children with varying diseases. Further research can be strengthened with the education of parents about the importance of enrolling their children in studies that utilize telemedicine services to improve adherence to care management plans and sustainability of the care model. While the benefit for a limited set of diseases is apparent, the effects of telemedicine on patient care and clinical outcomes need to be examined further for a wider range of conditions. By filling this gap in research, health care providers will find opportunities for greater utilization of telemedicine in their health systems.

Based on our findings, there are a wide variety of ways in which telehealth can be used effectively in a health system. Our brief report covers a limited scope of the types of services and devices that are being effectively used for RPM and telemedicine in pediatrics. These include CGM of pediatrics with type 1 diabetes, home monitoring of cardiovascular implantable devices, remote robotic telemedicine in the NICU, and remote consultation and diagnosis. We also presented closed-loop insulin delivery without remote monitoring and noncontact heart rate monitoring of infants in the NICU. The results of our systematic literature review may shed more light on potential research areas or adoption decisions by summarizing some of the more innovative and emerging telehealth capabilities being used throughout pediatric and neonatal health systems. However, this scoping review may help health care providers to remain current with the large plethora of emerging technologies and trends.

Our findings presented in this paper are also limited to studies in developed countries. One application has been reviewed in Malawi, Africa, a developing country. While this study was not necessarily relevant to the scope of this brief review, it may be important for future research and telehealth applications. In developing countries, access to a quality internet connection is rare, yet in larger cities, it is becoming more widely utilized by health systems and hospitals. Effective telemedicine consultations require high-quality equipment with appropriate internet connection and strong service coordination [18,23]. In Malawi, Africa, there are a total of 4 pathologists throughout the country, serving a population of 14 million [23]. The Queen Elizabeth Central Hospital in Malawi connected with a highly qualified hospital in Newcastle, United Kingdom, to obtain a speedy and efficient diagnosis of pediatric oncology cases. If the hospital in Malawi had waited for local diagnosis, they could spend anywhere from 3 weeks to 4 months waiting, whereas remote telepathologists were able to send diagnostic information within 24 hours; this is critical time for patients with oncological concerns, especially in resource-poor settings [23].

Some other aspects of RPM and telemedicine that have not been addressed in this report, but will be addressed in future reports, are cost savings to patients, families, and hospitals; the role of telesupport and telepresence between clinicians and providers; telediagnosis of a variety of diseases and medical conditions; and the importance of telemedicine in improving patient, family, and provider satisfaction.

### Conclusions

Despite the limited applications of telemedicine in pediatric and neonatal settings, current technologies show promise in several domains. Small sample size continues to be the main limitation of telemedicine studies in pediatrics. Continued research in telemedicine and RPM applications to a wider range of conditions will further emphasize the need for emerging trends in pediatric health systems. The information presented can inform health care providers of the most widely accepted forms of telemedicine for patients and their families and of the telemedicine that is most cost efficient for health systems. While the focus of this report is on RPM, we have presented some research studies that can inform providers about the importance of data sharing of remote monitoring data between hospitals. Continued reports of findings from this scoping literature review will educate key informants about the importance of telemedicine for pediatric populations and their families.

## Abbreviations

CGM: continuous glucose monitoring
ECG: electrocardiograph
NICU: neonatal intensive care unit
PPG: photoplethysmography
RPM: remote patient monitoring
RTP: robotic telepresence
